# *In-Vivo* Detection and Tracking of T Cells in Various Organs in a Melanoma Tumor Model by ^19^F-Fluorine MRS/MRI

**DOI:** 10.1371/journal.pone.0164557

**Published:** 2016-10-13

**Authors:** Christine Gonzales, Hikari A. I. Yoshihara, Nahzli Dilek, Julie Leignadier, Melita Irving, Pascal Mieville, Lothar Helm, Olivier Michielin, Juerg Schwitter

**Affiliations:** 1 Division of Cardiology, Lausanne University Hospital (CHUV), Lausanne, Switzerland; 2 Institute of Physics of Biological Systems, Ecole Polytechnique Fédérale de Lausanne (EPFL), Lausanne, Switzerland; 3 Molecular Modeling Group, Swiss Institute of Bioinformatics (SIB), Lausanne, Switzerland; 4 Ludwig Branch for Cancer Research of the University of Lausanne, Epalinges, Switzerland; 5 Institut des Sciences et Ingénierie Chimiques, Ecole Polytechnique Fédérale de Lausanne (EPFL), Batochime, Lausanne, Switzerland; 6 Department of Oncology, Lausanne University Hospital (CHUV), Lausanne, Switzerland; 7 Cardiac Magnetic Resonance Center, Lausanne University Hospital (CHUV), Lausanne, Switzerland; Centre National de la Recherche Scientifique, FRANCE

## Abstract

**Background:**

^19^F-MRI and ^19^F-MRS can identify specific cell types after in-vitro or in-vivo ^19^F-labeling. Knowledge on the potential to track in-vitro ^19^F-labeled immune cells in tumor models by ^19^F-MRI/MRS is scarce.

**Aim:**

To study ^19^F-based MR techniques for in-vivo tracking of adoptively transferred immune cells after in-vitro ^19^F-labeling, i.e. to detect and monitor their migration non-invasively in melanoma-bearing mice.

**Methods:**

Splenocytes (SP) were labeled in-vitro with a perfluorocarbon (PFC) and IV-injected into non-tumor bearing mice. In-vitro PFC-labeled ovalbumin (OVA)-specific T cells from the T cell receptor-transgenic line OT-1, activated with anti-CD3 and anti-CD28 antibodies (T_act_) or OVA-peptide pulsed antigen presenting cells (T_OVA-act_), were injected into B16 OVA melanoma-bearing mice. The distribution of the ^19^F-labelled donor cells was determined in-vivo by ^19^F-MRI/MRS. In-vivo ^19^F-MRI/MRS results were confirmed by ex-vivo ^19^F-NMR and flow cytometry.

**Results:**

SP, T_act_, and T_OVA-act_ were successfully PFC-labeled in-vitro yielding 3x10^11^-1.4x10^12 19^F-atoms/cell in the 3 groups. Adoptively transferred ^19^F-labeled SP, T_OVA-act_, and T_act_ were detected by coil-localized ^19^F-MRS in the chest, abdomen, and left flank in most animals (corresponding to lungs, livers, and spleens, respectively, with highest signal-to-noise for SP vs T_OVA-act_ and T_act_, p<0.009 for both). SP and T_act_ were successfully imaged by ^19^F-MRI (n = 3; liver). These in-vivo data were confirmed by ex-vivo high-resolution ^19^F-NMR-spectroscopy. By flow cytometric analysis, however, T_OVA-act_ tended to be more abundant versus SP and T_act_ (liver: p = 0.1313; lungs: p = 0.1073; spleen: p = 0.109). Unlike ^19^F-MRI/MRS, flow cytometry also identified transferred immune cells (SP, T_act_, and T_OVA-act_) in the tumors.

**Conclusion:**

SP, T_act_, and T_OVA-act_ were successfully PFC-labeled in-vitro and detected in-vivo by non-invasive ^19^F-MRS/MRI in liver, lung, and spleen. The portion of ^19^F-labeled T cells in the adoptively transferred cell populations was insufficient for ^19^F-MRS/MRI detection in the tumor. While OVA-peptide-activated T cells (T_OVA-act_) showed highest infiltration into all organs, SP were detected more reliably by ^19^F-MRS/MRI, most likely explained by cell division of T_OVA-act_ after injection, which dilutes the ^19^F content in the T cell-infiltrated organs. Non-dividing ^19^F-labeled cell species appear most promising to be tracked by ^19^F-MRS/MRI.

## Introduction

Cell tracking by magnetic resonance imaging (MRI) is an emerging method to visualize and monitor labeled cells after transplantation non-invasively and without the use of ionizing radiation. Recently, ^19^F-fluorine-MRI has been used to detect and track well-defined cell populations [1–7]. Because of the effective absence of ^19^F background signal in the body, any^19^F signal detected after injection of a ^19^F compound is unequivocally produced by this injected compound. As the MR signal is directly proportional to the amount of ^19^F nuclei present in the tissue, it can be related to a reference of known ^19^F concentration, rendering this technique quantitative [3, 4]. Moreover, these compounds are not limited by signal decay over time and therefore the time window for their detection can last several days. Finally, the ^19^F signal can be merged with conventional ^1^H-MRI images to identify its exact anatomic location and to add information on structure, function, and tissue characteristics. Direct IV injection of emulsions containing ^19^F-based perfluorocarbons (PFC) has been performed in different rodent models for angiography [8] and to detect non-invasively inflammation in myocardial infarction [5, 9], cerebral ischemia [5], myocarditis [6], pneumonia [10], atherosclerosis [11], arthritis [12] and tumors infiltrated by macrophages [13]. Distinctively, defined cell populations such as dendritic cells [1], T cells [3, 4, 14, 15], or mesenchymal stem cells [16] were tracked non-invasively in rodents by ^19^F-MRI or ^19^F-MR spectroscopy (^19^F-MRS) after their in-vitro ^19^F-labeling. Recently, clinical ^19^F-MRI cell detection using labeling by PFC has also been described in patients with colorectal adenocarcinoma in order to detect autologous immunotherapeutic dendritic cells [7]. This technique could therefore be applied to detect tumor cells as well as to monitor adopted cell transfer cancer therapies.

In recent years adoptive cell transfer therapies using ex-vivo activated T cells have undergone intensive testing [17, 18], and various types of T cells have been used for adoptive immunotherapy. It is essential to know whether the administered T cells reach their target and this is currently assessed by biopsies, which are invasive and not practical for all patients [18]. Also, with a biopsy-based approach the total amount of T cells in a tumor, their distribution, and the kinetics of cell fluxes are difficult to assess. Non-invasive visualization of the trafficking of administered T cells could potentially allow one to predict responsiveness to these therapies. Therefore, a reliable non-invasive imaging method to monitor anti-tumor cell traffic is highly desirable. Moreover, as T cells with specific anti-tumor properties can migrate to and infiltrate tumor tissue by recognizing tumor antigens [19], they could, in principle, be used as a probe to detect tumor cells at metastatic sites when labeled with PFCs.

In the present study the migratory behavior of 3 different cell populations was tracked by means of non-invasive ^19^F-MRS and ^19^F-MRI and compared with invasive flow cytometry analyses and high-resolution in-vitro ^19^F-NMR. Initially, splenocytes (SP) were labeled in-vitro by a PFC to test the feasibility of non-invasive in-vivo tracking by ^19^F-MRS and ^19^F-MRI in control mice. SP represents a heterogeneous cell population comprising not only T cells (both CD8^+^ and CD4^+^, naïve, effector, memory and regulatory cells), but also B cells and antigen presenting cells (including dendritic cells, monocytes, macrophages and myeloid cells). The activated T cell populations, T_OVA-act_ and T_act_, whereas, are mostly CD8^+^ and these cytotoxic lymphocytes express one unique T cell receptor (TCR) called OT-1. To distinguish how the T cells were activated and expanded in-vitro, we named “T_act_” the T cells that were stimulated with anti-CD3 and anti-CD28 antibodies, and “T_OVA-act_” the cells derived from single-cell suspensions of dissociated spleens stimulated with the specific OVA_257-264_ peptide. The OVA_257-264_ antigen was used as a tumor-specific antigen in the current study, and T_OVA-act_ and T_act_ were produced from OT-1 mice expressing only the TCR OT-1 specific for K^b^-OVA_257-264_ which is expressed at the surface of B16-OVA tumor grafted on recipient mice.

Splenic-derived OT-1 CD8^+^ T cells, stimulated either by OVA-peptide (= T_OVA-act_) or by anti-CD3 and anti-CD28 antibodies (= T_act_) will expand and differentiate into various states including central memory (T_CM_), effector memory (T_EM_) and terminally differentiated, short-lived effector T cells (T_E_). Importantly, the newly activated T cells will also maintain a high state of proliferation for several days. While T_E_ cells are typically found in peripheral tissue and provide a critical first line of defense to foreign antigen, T_CM_ cells migrate to areas of secondary lymphoid organs, and compared to naive T_N_ cells have a higher sensitivity to antigen stimulation. T_EM_ tend to home to inflamed tissues, and have a more rapid effector function as compared to T_CM_ [20]. Activated tumor-antigen specific T_OVA-act_ and T_act_ were labeled in-vitro (by the same PFC as used for SP) to test for non-invasive in-vivo tracking by ^19^F-MRS and ^19^F-MRI in mice bearing a B16-OVA tumor. Accordingly, the aim of the study was to develop a reproducible protocol for the in-vitro ^19^F-labeling of the three cell groups, to determine the detection limits of ^19^F-MRS and ^19^F-MRI for the in-vivo detection of these cells, and to test this application in B16-OVA tumor bearing mice.

## Materials and Methods

### Tumor Cells

B16-OVA melanoma cells (Ludwig Branch for Cancer Research, Lausanne) were maintained in Dulbecco's modified Eagle's medium (GIBCO Invitrogen, Grand Island, NY) supplemented with 10% heat-inactivated fetal bovine serum (GIBCO Invitrogen) and penicillin-streptomycin (GIBCO Invitrogen).

### Abs and reagents

Fluorescent antibodies against mouse CD3, CD4, CD8, CD19, CD11b, CD44, anti-CD62L, PD-1, CD127, CD45.2, CD107a and IFNɣ were purchased from eBioscience (San Diego, CA). Purified anti-mouse CD3 and anti-mouse CD28 were from Biolegend (San Diego, CA). The OVA peptide (SIINFEKL) was produced by the Protein and Peptide Chemistry Facility located in the Department of Biochemistry of the University of Lausanne. FITC-conjugated or unconjugated ^19^F-based perfluorocarbons (PFC) were purchased from Celsense (Pittsburgh, PA).

### Animals

**Fig 1:**
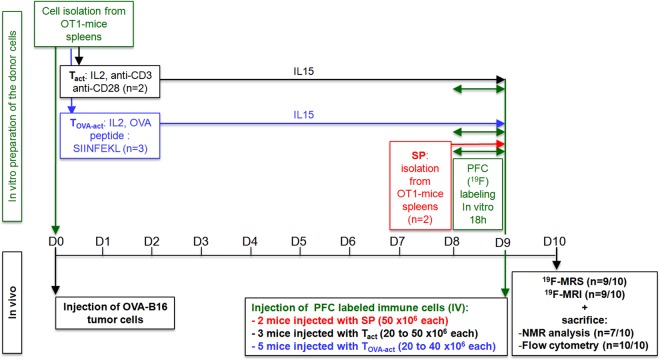
In-vivo protocol description. Overview of the time scale of the different experimental procedures. At day 0 (D0) eight CD45.1 C57BL/6 mice received 10^6^ B16-F10 melanoma cells by subcutaneous injection in order to induce a malignant melanoma. On the same day SP were prepared from OT-1 mice and two different protocols were applied to generate T_act_ or T_OVA-act_ (as described in Materials and Methods, T cells isolation and activation section). At day 8 (D8), PFC was added in the cell culture medium for 18h in order to label SP, T_act_ and T_OVA-act_ with ^19^F. Then, at day 9 (D9) the ^19^F-labeled cells were injected IV: 2 control mice (with no tumors) received 50 x 10^6^ SP, 3 mice received 20 to 50 x 10^6^ T_act_ and 5 mice received 20 to 40 x 10^6^ T_OVA-act_. Finally, 9 mice were imaged at day 10 (D10; 1 T_act_ injected mouse was not imaged) and all mice were immediately sacrificed for subsequent analysis of the organs (liver, lungs, spleen and tumor) by flow cytometry (all mice) and high resolution in-vitro NMR spectroscopy (2 SP injected mice, 3 T_OVA-act_ injected mice and 1 T_act_ injected mouse). The study protocol was performed in a total of 10 animals. In black: in-vivo part; in blue: cell preparation.

All animal procedures were approved by the animal ethics committee (SCAV: Service de la Consommation et des Affaires Vétérinaires, Epalinges, Switzerland). All MR examinations were performed under ketamine-medetomidine anesthesia, and all efforts were made to minimize suffering. Mice were maintained under specific pathogen-free conditions. Ovalbumin-specific TCR transgenic (OT-1) mice were used to produce SP (described below). OT-1 mice were on a RAG1^-/-^ background. CD45.1 C57BL/6 mice were used as recipients for adoptive transfer (described below). Ten days prior to adoptive transfer, tumors were implanted subcutaneously and dorsolaterally with inoculations of 10^6^ B16-F10-OVA melanoma cells in 50 μl saline in CD45.1 C57BL/6 mice [21]. The in-vivo protocol is depicted in Fig 1.

### T cell isolation and activation

Spleens from CD45.2^+^ OT1 mice were removed aseptically and homogenized by passing through a cell strainer (40μm). Red blood cells were lysed by the addition of a buffered ammonium chloride solution. The nucleated remaining cells (SP) were resuspended in complete medium (RPMI-1640 medium with 10% FBS, 100 μg/ml each of streptomycin and penicillin, 10 mM HEPES and supplemented with 2-mercaptoethanol) and two different protocols were applied to produce either T_act_ or T_OVA-act_. The SP population was used immediately after isolation for in-vitro FITC-conjugated or un-conjugated ^19^F-labeling and dead cells were eliminated with Ficoll (GE Healthcare) prior to injection into CD45.1^+^ C57BL/6 mice. T_act_ were obtained by stimulation of the SP population with recombinant murine IL2 (20 ng/ml), anti-mouse CD3 (500 ng/ml) and anti-mouse CD28 (0.1 μg/ml) for 2 days. T_OVA-act_ were obtained by stimulation of the SP with the OVA peptide (SIINFEKL; 2 μg/ml) for 2 days in the presence of recombinant murine IL2 (20 ng/ml). In both protocols, two days after stimulation, clusters were formed and harvested to form a single-cell suspension. Dead cells were eliminated with Ficoll and cells were seeded in complete medium containing recombinant human IL15 at 20 ng/ml. Medium was changed every 2 days for one week.

### Cell Labeling

SP, T_act_, and T_OVA-act_ were labeled in-vitro with Cell Sense (CS-1000), a ^19^F-based MR imaging agent. Cell Sense is an aqueous colloidal suspension (= nanoemulsion) of a perfluoropolyether perfluorocarbon polymer (PFC), having total fluorine content of 145 mg/mL (Celsense Inc., Pittsburg, PA, USA). The average nanoemulsion droplet size is 180 nm. It is formulated with excipients that facilitate PFC uptake into all cell types, regardless of their ability to phagocytose. The PFC used in Cell Sense is stable at low pH [22]. SP, T_act_, and T_OVA-act_ were also labeled in-vitro with FITC conjugated PFC. In all conditions, PFC was added to the cell culture medium at a concentration of 10 mg/mL and incubated with the SP, T_act_, or T_OVA-act_ for 18 hours at 37°C, 5% CO_2_ (SP n = 5; T_act_ n = 14; T_OVA-act_ n = 10). After this incubation period, the cells were washed three times with PBS and counted.

### High resolution in-vitro ^19^F-NMR spectroscopy of labeled cells

In order to measure the mean ^19^F content present in the cells after labeling, quantitative ^19^F NMR measurements were performed in lysed cell pellets. A known number of labeled cells (~3x10^6^) were spun down, resuspended in 250 μl of 1% Triton X100 v/v in PBS to lyse the cells. The cell lysate was mixed with 250 μl of a calibrated ^19^F reference solution, trifluoroacetic acid (TFA) at 0.1% v/v in D_2_O, and placed in a 5 mm NMR borosilicate tube. The ^19^F NMR measurements were performed using a Bruker AVANCE III HD 400 MHz (9.4 T) NMR spectrometer (Bruker BioSpin AG, Fällanden (ZH), CH). The average ^19^F-fluorine content per cell was calculated from the ratio of the integrated areas of the TFA and PFC ^19^F spectra, normalized to the total cell number in the lysate. PFC ^19^F spectra, acquired with 256 scans and processed with a line broadening of 5 Hz, contain several peaks with a major one located at -93 ppm and the TFA peak at -75 ppm. These two peaks were used for quantitative calculations.

### Functional assay of PFC-FITC labeled cells

One hundred thousand SP, T_act_, and T_OVA-act_ were cocultured with 50x10^5^ B16-OVA tumor cells in the presence of anti-CD170a antibody and Golgi Stop reagent (BD Biosciences, San Jose, CA). After 5 hours of incubation at 37°C, cells were washed and stained with fluorescent anti-CD3 and anti-CD8 antibodies at 4°C for 20 minutes. Following fixation and permeabilization, the cells were stained with anti-IFNɣantibody and analyzed on a FACS LSRII (BD Biosciences) and BD FACS Diva software.

### Cytotoxicity assay of PFC-FITC labeled cells

Fifty thousand SP, T_act_, and T_OVA-act_ were co-cultured with 25x10^5^ B16-OVA tumor cells in the presence of Cytotox red reagent (Essen Bioscience, Ann Arbor, Michigan), according to the manufacturer’s instructions. Images were acquired every 2 hours with the Incucyte Zoom System (Essen Bioscience) and analyzed with its software.

### Phantom experiments

Different dilutions of TFA (0.6M, 0.5M, 0.4M, 0.3M, 0.2M, 0.1M) in 0.3M NaCl were prepared in agarose gel for imaging. ^1^H images were acquired with a 9.4T spectrometer (Varian, Palo Alto, CA) using a gradient echo sequence (repetition time (TR) 13.2 ms, echo time (TE) 2.4 ms, signal averages 8, matrix 128×128, field of view 18×18 mm^2^, 3 slices with a slice thickness of 2 mm, total acquisition time 13.5 s). Next, for the ^19^F acquisitions at the same locations of the ^1^H images, a fast spin echo sequence was used with TR = 500 ms, TE = 3.7 ms; echo-train length 4, signal averages 960 scans, matrix 32x32, field of view 18×18mm^2^, slice thickness 2 mm, and a total acquisition time of 64 minutes.

### Adoptive transfer of T cells

The study protocol is shown in Fig 1. PFC labeled SP-CD45.2^+^ (50 ×10^6^) suspended in 250 μl NaCl solution were injected i.v. into two control (no tumor implanted) recipient CD45.1^+^ C57BL/6 mice. PFC labeled T_act_ or T_OVA-act_ (CD45.2^+^; 20 to 50 ×10^6^) suspended in 250 μl NaCl solution were injected i.v. into recipient CD45.1^+^ C57BL/6 mice that were implanted with B16-F10-OVA melanoma cells 9 days before. The injection of CD45.2^+^ immune cells into CD45.1^+^ C57BL/6 recipient mice allows discriminating injected immune cells from host cells.

### ^1^H and ^19^F-MRI

The day after adoptive transfer (24h to 36h post injection, Fig 1) mice were anesthetized with intraperitoneal injection of ketamine: medetomidine (75 mg/kg: 0.1 mg/kg). This anaesthetic combination was chosen to avoid any isoflurane ^19^F-MR background signal resulting from its accumulation in the fat pads [23]. The body temperature was monitored with a rectal probe (SA Instruments, Stony Brook, NY) and kept constant at 37.0°C by using tubing with circulating warm water. The animals were placed under a custom-designed 18-mm diameter quadrature surface coil tunable to both the ^1^H and ^19^F frequencies (400.2 and 376.6 MHz, respectively). To acquire coil-localized spectra of ^19^F (128 scans) the coil was positioned at 4 different places, the chest, abdomen, left flank, and the right thigh to cover primarily the liver, the lungs, the spleen, and the tumor, respectively.

In 3 mice, the ^19^F spectroscopic signal was deemed sufficient for ^19^F-MRI (signal-to-noise ratio (SNR) >200). In these mice a stack of 6 axial ^1^H images of the liver was acquired with a gradient echo sequence (repetition time (TR) 29.7 ms, echo time (TE) 1.9 ms, signal averages 4, matrix 128×128, field of view 30×30 mm^2^, slice thickness 2 mm, total acquisition time 15.2 s). Next, a stack of axial ^19^F images was acquired at the identical position as the ^1^H images using a fast spin echo sequence (TR = 500 ms, TE = 3.7 ms; echo-train length 4, signal averages 960, matrix 16x16, field of view 30×30mm^2^, slice thickness 2 mm, total acquisition time 32 minutes).

### Organ collection

Immediately after the MRI session the mice were euthanized by cervical dislocation to harvest the liver, lungs, spleen and tumor. A single-cell suspension was then prepared from the different organs using a cell strainer (70 μm) and RPMI medium. The different cell suspensions were washed once with RPMI and then split into two groups with 5% of the cell suspension used for flow cytometry analyses and 95% for high resolution ex-vivo spectroscopy ^19^F-NMR analyses.

### High resolution ex-vivo ^19^F-NMR spectroscopy of excised organs

The ^19^F NMR measurements were performed on the cell suspensions prepared from the different organs described above. Ninety-five percent of the cell suspensions from liver, lungs, spleen and tumor were centrifuged and then resuspended into 250 μl of 1% Triton X100 v/v in PBS to lyse the cells. The cell lysates were then mixed with 250 μl of TFA 0.1% v/v in D_2_O (calibrated ^19^F reference solution), and placed in a 5 mm NMR borosilicate tube. The acquisition method used was described previously in the “High resolution in-vitro ^19^F-NMR spectroscopy of labeled cells” section.

### Statistical analyses

Values are given as means ± standard deviation. Analyses of differences between groups were performed using unpaired Student’s t-test and one-way analysis of variance (ANOVA) where appropriate (GraphPad Prism software).

## Results

### In vitro labeling and function of immune cells

**Fig 2:**
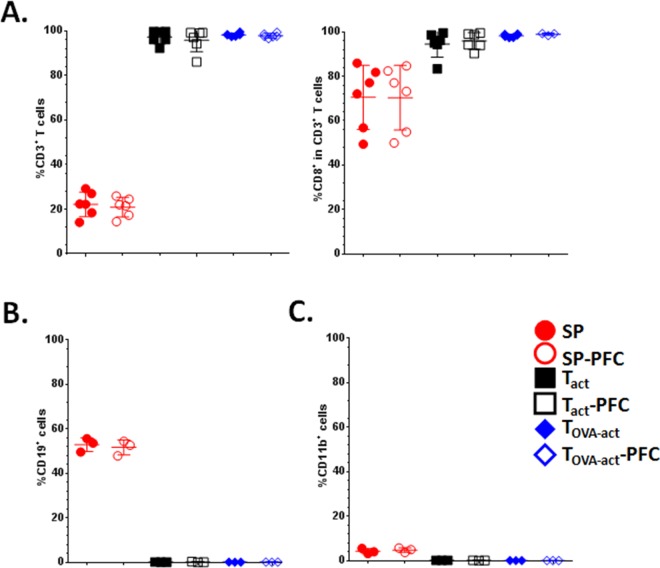
Phenotype of total splenocytes (SP), anti-CD3/anti-CD28 activated (T_act_) and Ova-peptide activated (T_OVA-act_) splenocytes from OT-1 cells, before and after PFC labelling. (**A**) Percentage of CD3^+^ T cells in the total population and proportion of CD8^+^ T cells in CD3^+^ population. (**B**) and (**C**) Percentage of CD19^+^ and CD11b^+^ cells in the total population. (n = 3 for each group of cells)

The 3 cell groups were in-vitro labeled or not with FITC conjugated or unconjugated ^19^F -PFC in order to assess cell labeling efficiency, and to compare cell viability, phenotype and T cell function. After 18 hours of incubation with PFC the cells were stained by Trypan blue exclusion assay to evaluate the potential cytotoxicity due to labeling. For the 3 cell groups (SP, T_act_ and T_OVA-act_), the amount of dead cells after PFC incubation was comparable to the untreated control condition (difference when compared to untreated cells of the same type: 0.5%, 7% and 3% for SP, T_act_, and T_OVA-act_, respectively). This result shows that the PFC-based protocol safely labels these cells in-vitro. Moreover, PFC labeling does not affect the proportion of cell populations, no difference was observed after FITC-conjugated PFC staining (Fig 2). SP are composed of ~25% of CD3^+^ T cells (Fig 2A), ~55% of CD19^+^ non-T cells (Fig 2B), ~5% of CD11b^+^ non-T cells (Fig 2C), whereas T_act_ and T_OVA-act_ are composed of only CD3^+^ T lymphocytes (Fig 2A). In CD3^+^ T cells, almost all cells are CD8^+^ T cells (Fig 2A) because they are derived from transgenic OT-1 mice.

**Fig 3:**
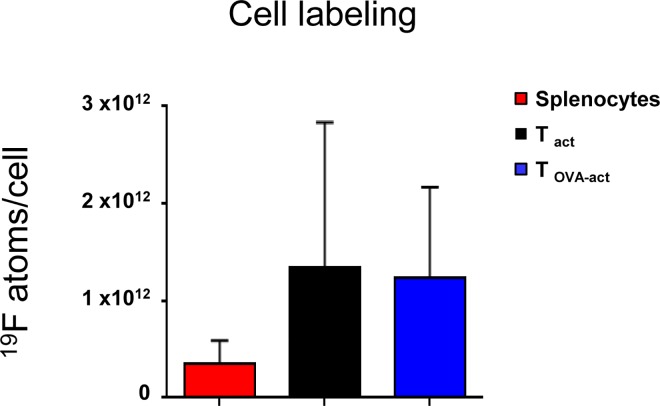
In vitro cell labeling. ^19^F content (number of ^19^F atoms per cell) in SP, T_act_ and T_OVA-act_ after incubation with PFC for 18h. Cellular loading was determined in a known number of cells by using an internal reference (TFA) with a known number of ^19^F atoms.

Fig 3 depicts the ^19^F content (i.e. the number of ^19^F atoms per cell, for the SP, T_act_, and T_OVA-act_ cells) after 18h of incubation with the PFC agent quantified by high-resolution ex-vivo ^19^F NMR. The mean ^19^F content per cell was similar for the 3 cell groups (Fig 3, overall-p = 0.72).

**Fig 4:**
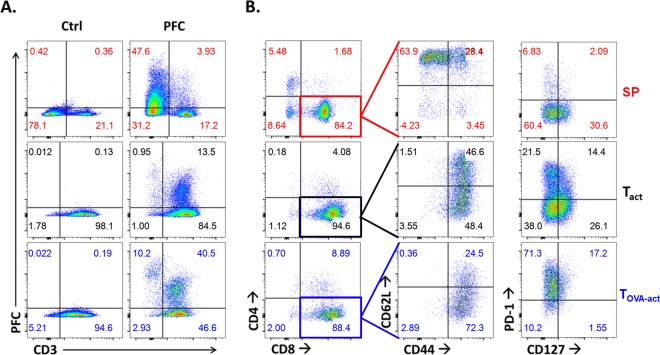
(**A**) PCF labelling of total splenocytes (SP), anti-CD3/anti-CD28 activated (T_act_) and Ova-peptide activated (T_OVA-act_) splenocytes from OT-1 cells. (**B**) Phenotype of CD3^+^ T cells: CD3^+^CD8^+^ T cells were analyzed by flow cytometry for CD44, CD62L, CD127 and PD-1 expression. By this flow-cytometric analysis, the following phenotypes could be identified in the CD3^+^CD8^+^ T-cell population: CD62L^+^CD44^-^ (naive T cells), CD62L^+^CD44^+^ (memory T cells) and CD62L^-^CD44^+^ (effector T cells). n = 3 for each group of stimulation. Data are representative of 3 independent experiments.

In the SP group, the non-T cells take up the most PFC (44.93% ± 3.76% of CD3^-^PFC^+^ vs 3.70% ± 0.21% of CD3^+^PFC^+^, n = 3, Fig 4A). In the T_act_ and in T_OVA-act_ groups the percentage of PFC^+^ cells are 13.70% ± 0.61% and 28.67% ± 7.30%, respectively, n = 3 (Fig 4A). Non-T cells were preferentially labeled by PFC and T_OVA-act_ presented a 2-fold higher labeling than T_act_. In the SP group, the majority of lymphocytes are naïve T cells (54,96% ± 5.91% of CD62L^+^CD44^-^ cells in CD3^+^ cells, n = 3) with weak expression of PD-1, a major inhibitory receptor regulating T-cell exhaustion (Fig 4B). After stimulation, T_act_ and T_OVA-act_ present the phenotype of central memory (CD44^+^CD62L^+^) and effector memory (CD44^+^CD62L^-^) cells (Fig 4B). The proportion of these 2 populations is different in T_act_ and T_OVA-act_. OVA-peptide stimulation preferentially induces an effector memory (62.80% ± 6.25%, n = 3) rather than a central memory phenotype (34.13% ± 5.85%, n = 3). Conversely, antibody stimulation leads to a higher central memory (50.73% ± 5.29, n = 3) than effector memory phenotype (43.33% ± 4.72, n = 3). Moreover OVA-peptide stimulation is stronger than antibody stimulation in our experimental conditions as it induces an overexpression of PD-1 (88.83% ± 0.82%, n = 3) compared to T_act_ cells (46.63% ± 3.62%, n = 3). All of these phenotypic differences may impact PFC uptake.

**Fig 5:**
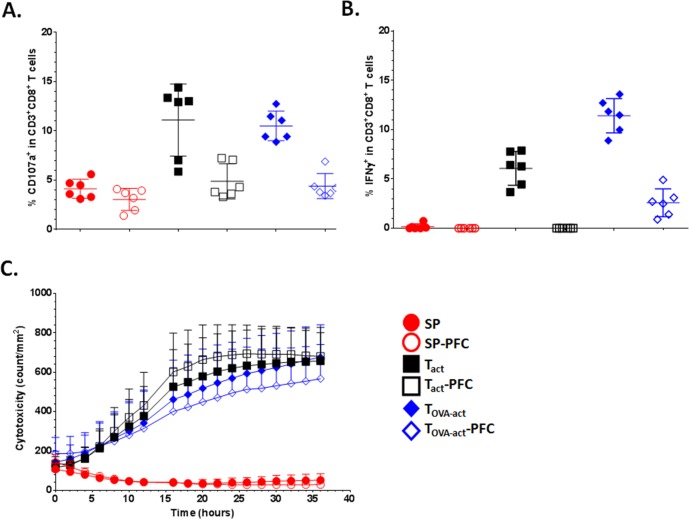
PCF labelling does not affect the function of total splenocytes (SP), anti-CD3/anti-CD28 activated (T_act_) and Ova-peptide activated (T_OVA-act_) splenocytes from OT-1 mice. CD107a upregulation (**A**) and IFNɣ production (**B**) by CD3^+^CD8^+^ T cells were analyzed after co-culture of corresponding cells with B16-OVA mouse melanoma cells, used as antigen presenting cells. (**C**) Cytotoxicity of corresponding cells in same conditions was measured by the incorporation of red reagent in dead cells. The acquisition was done by Incucyte microscope. (n = 3 for each group of stimulation)

Finally to determine the impact of PFC on T cell function, we performed a series of experiments before and after PFC-labeling. As expected, SP showed a weak response due to the small proportion of CD3^+^ T lymphocytes (Fig 5). PFC-labeling induced a decrease of response in CD107a upregulation (Fig 5A) and IFNɣ (Fig 5B) secretion assays, but did not impact the cytotoxic capacity of T cells (Fig 5C). Hence, PFC-labeled T cells are able to recognize and kill their target.

### Limit of detection of ^19^F-PFC-labeled immune cells by ^19^F-MRI

**Fig 6:**
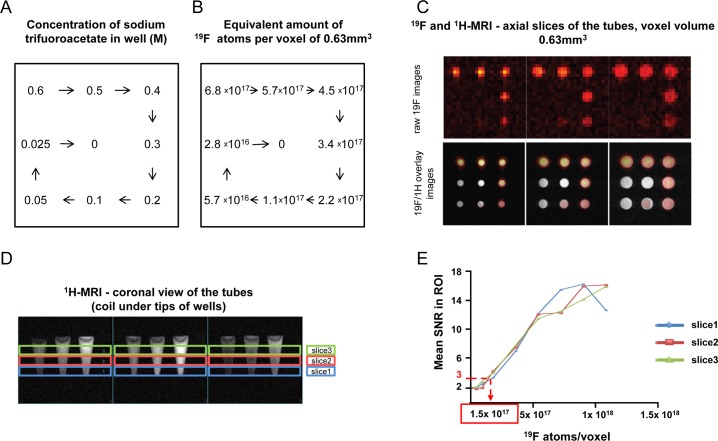
Limit of detection: Phantom with TFA. (A) Concentration in M of TFA per well. (B) Equivalent number of ^19^F atoms per well. (C) Axial view of the tubes. Upper panels: ^19^F images acquired; lower panels: ^19^F and ^1^H overlaid images. Voxel volume: 0.63mm^3^. (D) Coronal view of the tubes, ^1^H images. (E) Mean SNR in the region of interest (ROI) with respect to ^19^F atoms/voxel. Each line corresponds to the acquisition of a slice as shown in panel D.

In order to determine the limit of detection of the method, a phantom experiment was performed using different TFA dilutions (Fig 6A and 6B). Both ^1^H and ^19^F images were acquired for each dilution (Fig 6C). Under these conditions the limit of detection for ^19^F-MRI was 1.5 x 10^17 19^F spins (at a SNR level of 3), which would correspond to 150’000 cells per voxel of 0.63 mm^3^ assuming a cell labeling of 10^12 19^F atoms/cell (Fig 6E) corresponding to 238’000 cells per μl.

### In-vivo detection of ^19^F-MRI signal

**Fig 7:**
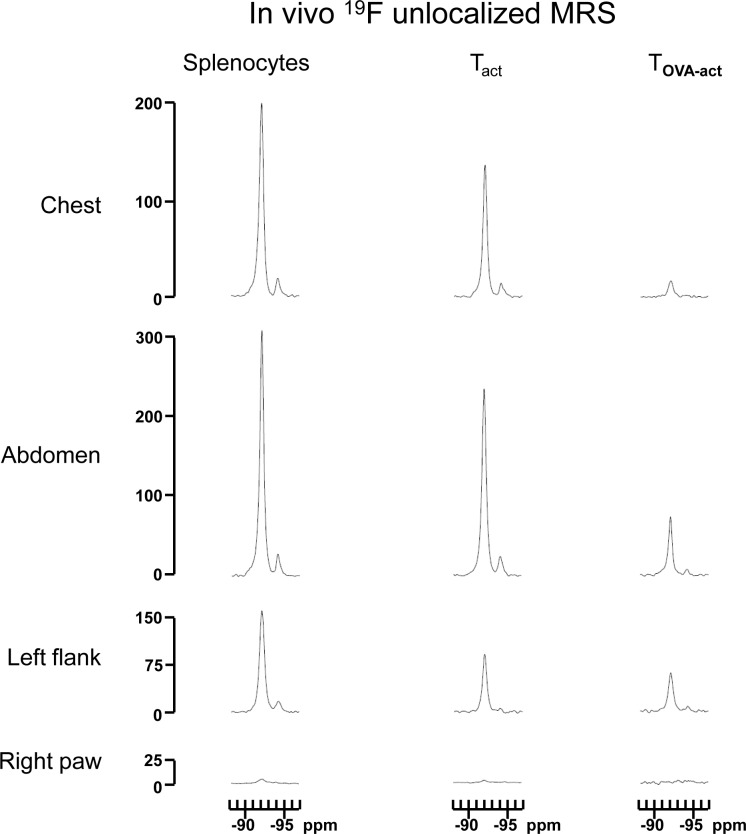
MRS spectra. Representative ^19^F spectra of coil-localized spectroscopy acquired in-vivo in 4 different regions (chest, abdomen, left flank, and right thigh) of the mice injected with SP, T_act_ and T_OVA-act_. Highest signals were measured in the chest and the abdomen while no reliable signal was detected in the right thigh. ^19^F-MRI of SP and T_act_ injected animals are depicted in Fig 9B (middle and lower panels).

**Fig 8:**
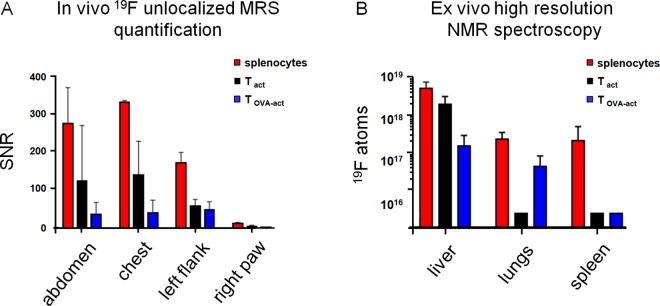
In-vivo coil-localized spectroscopy and ex-vivo high resolution NMR spectroscopy. (A) SNR (mean ±SD) of ^19^F coil-localized spectroscopy acquired in the four different regions of the mice. In SP injected mice higher ^19^F-signals were observed compared to the T_act_ and T_OVA-act_ injected mice both in the abdomen and the left flank regions (p<0.05, one way ANOVA). (B) Ex-vivo ^19^F content (mean ±SD) in liver, lung, and spleen. No ^19^F signal was detected in the tumor. The internal reference (TFA) contained a known number of ^19^F atoms. There is a higher ^19^F load in the liver in SP injected compared to T_OVA-act_ injected mice (p = 0.05: all other comparisons are non-significant). Note logarithmic scale of y-axis in B.

In order to follow the migration of the injected immune cells in-vivo, ^19^F-MRS was performed in different anatomic areas (chest, abdomen, left flank and right thigh) of the mice injected with ^19^F labeled SP, T_act_, and T_OVA-act_ cells. These acquisitions were performed 24h after adoptive cell transfer. Fig 7 shows examples of ^19^F coil-localized spectra and Fig 8A. No signal was detectable in the right thigh (corresponding to the tumor area) whereas ^19^F signal was detectable in the chest, abdomen, and left flank of most of the animals, corresponding primarily to the lungs, the liver, and the spleen, respectively. The ^19^F signal measured in the abdomen and in the left flank was significantly higher in the SP injected group compared to the other groups (one way ANOVA, p = 0.0083 and p = 0.0076 respectively, Fig 8).

**Fig 9:**
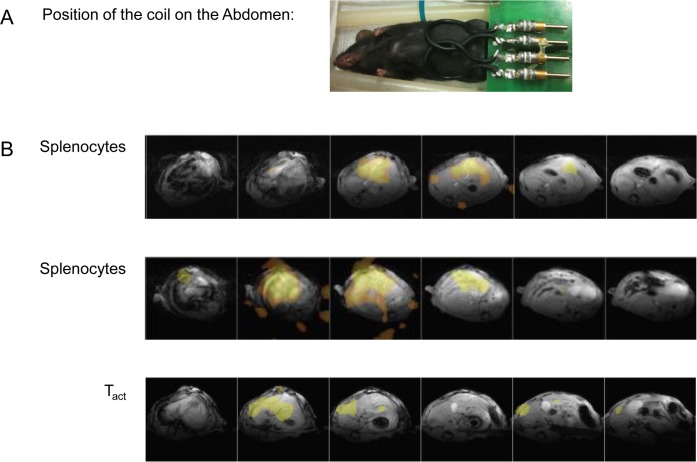
In-vivo imaging. (A) Image showing the position of the surface coil that covers the abdomen (liver and part of the lungs) used for ^19^F-MRS, ^19^F-MRI, and ^1^H-MRI. (B) Representative axial MRI of mice that received either SP (upper and middle panels) or T_act_ (lower panels). The livers can clearly be identified. The ^19^F-MRS spectra of these animals (SP middle panels and T_act_ lower panels) are illustrated in Fig 4. No images were acquired for mice injected with T_OVA-act_. ^1^H-MRI in grey and ^19^F-MRI in orange. The total ^19^F content in the liver of the animals measured by ex-vivo ^19^F-MRS was 1.5 x 10^18^ and 1.2 x 10^18^ after SP injection, and 1.15 x 10^18^ after T_act_ injection (compare also Fig 8B).

Fig 9 shows representative images of ^19^F and ^1^H-MRI overlays with a strong ^19^F-signal in the liver of 3 animals, i.e. in which the spectroscopic ^19^F signal was deemed sufficient for ^19^F-MRI (SNR >200). As anesthesia duration was limited, the abdomen was the only area imaged.

### Ex-vivo high resolution ^19^F-NMR spectroscopy

A post-mortem in-vitro quantitative analysis of ^19^F-NMR spectra of different organs (liver, lungs, spleen, tumor) was performed as a reference for ^19^F organ content after administration of ^19^F labeled cells. The ^19^F content of the different homogenized organs is depicted in Fig 8B. ^19^F signal was consistently measured in the liver (in 6 out of 7 animals) as well as in the lungs (4 of 7) and occasionally in the spleen (1 of 7). No ^19^F signal was observed in the tumors of these animals. Moreover, the ^19^F measured signal was higher in SP injected mice compared to T_OVA-act_ injected mice (p<0.0001 in liver and not significant in lungs, ANOVA).

### Flow cytometry

**Fig 10:**
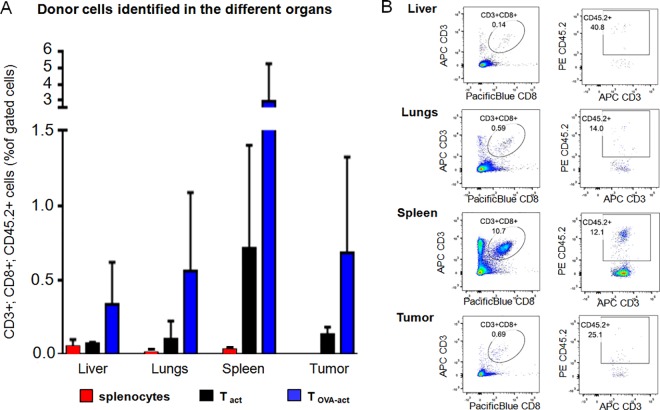
Flow cytometry analysis of the donor T cells (CD3+; CD8+; CD45.2+) found in the organs. (A) Amount of (CD3+; CD8+; CD45.2+) cells in the liver, lungs, spleen, and tumor. Cell suspensions from the organs were stained with monoclonal antibodies for CD3 and CD8 together with monoclonal antibody for CD45.2 to identify adoptively transferred cells. Gating was performed on CD3^+^; CD8^+^ cells with subsequent analysis of CD45.2^+^ (to differentiate the host cells (CD45.1^+^) from transplanted cells (CD45.2^+^)). Data are expressed in CD3^+^; CD8^+^; CD45.2^+^ cells as percent of gated cells. (B) Data from one representative T_act_ injected mouse are shown for the different organs analyzed.

In order to confirm the label tracking results from in-vivo ^19^F-MRS, in-vivo ^19^F-MRI, and ex-vivo ^19^F-NMR, the distribution of the donor T cells in the different organs was determined by flow cytometry according to their expression of CD3, CD8 and CD45.2. There was massive infiltration and proliferation of donor cells in a variety of peripheral tissues, including liver, spleen, and lungs (Fig 10). A small population of these donor cells (CD3^+^ CD8^+^ CD45.2^+^) was also found in the tumors, with no significant difference between the 3 cell types. However, contrary to what was observed with in-vivo and ex-vivo ^19^F-analyses, the amount of adoptively transferred SP was very low in all the organs analyzed and the amount of adoptively transferred T_OVA-act_ more abundant in the different organs compared to either SP or T_act_, although not to a significant degree (liver: p = 0.1313; lungs: p = 0.1073; spleen: p = 0.109).

## Discussion

In this study ^19^F-PFC was used to in-vitro label SP and activated T cells and to follow their migration in-vivo in B16-OVA-melanoma bearing mice using ^19^F-MRS and ^19^F-MRI.

### In-vitro labeling of immune cells and the detection threshold by ^19^F-MRI

SP, T_act_, and T_OVA-act_ were successfully labeled in-vitro, achieving similar ^19^F content per cell in the 3 populations ranging from 3 x 10^11^ to 1.4 x 10^12^ atoms/cell. Labeling of activated T cells is consistent with data published by Srinivas et al. reporting a ^19^F loading per cell of 1.7 ± 0.9 × 10^12 19^F/cell [4]. In the present study there are, however, some differences compared to the study of Srinivas. We used a commercially available PFC, while Srinivas et al. used a perfluorinated polyether emulsion prepared in their own lab, which required 3 days of incubation to label cells, in contrast to the 18h-incubation in the present protocol. Importantly, the labeling procedure used in the current work did not affect cell viability.

With an in-vitro phantom experiment we determined the detection threshold for ^19^F-MRI for in-vitro ^19^F-PFC-labeled cells. Approximately 150’000 cells with an assumed ^19^F loading of 10^12^ atoms/cell are detectable in a minimal voxel volume of 0.63mm^3^ (measured over 34 minutes at 9.4T at an SNR of 3).

### In-vivo and ex-vivo detection of in-vitro ^19^F-labeled immune cells by ^19^F-MRS and ^19^F-MRI

Initially, ^19^F-PFC labeled SP were tested in non-tumor bearing mice as a proof of concept. Using in-vivo ^19^F-MRS we detected the donor cells in the area of the abdomen, the chest, and the left flank corresponding mainly to the liver, the lungs, and the spleen, respectively (Fig 8A). These in-vivo data were confirmed post-mortem by in-vitro quantitative ^19^F-NMR, yielding a similar distribution of ^19^F signals as shown in Fig 8B. Then, in a next step, ^19^F-labeled T_act_ and T_OVA-act_ immune cells were adoptively transferred into B16-OVA tumor-bearing mice. As with SP cells, donor T_act_ and donor T_OVA-act_ were detected by in-vivo ^19^F-MRS in the areas of the liver, lungs, and spleen (Fig 8A) and these data were confirmed by ex-vivo quantitative ^19^F-NMR measurements (Fig 8B). As for in-vivo ^19^F-MRS, the post-mortem ^19^F-NMR detected highest ^19^F quantities in SP injected animals with most frequent positive findings in livers (6 of 7 animals) followed by lungs (4 of 7 animals) and the spleen (1 of 7 animals). Thus, in-vivo ^19^F-MRS allows for cell detection in agreement with the true ^19^F distribution in these organs. In addition, the same donor cells were successfully imaged in-vivo by ^19^F-MRI in the livers of 3 mice.

Our data show that following IV injection of in-vitro ^19^F-labeled cells, the majority of injected cells are trapped in the liver and the lungs (Fig 8B). It has previously been shown that a large fraction of IV-injected CD8^+^ T cells preferentially migrate into the interstitium of normal lungs [24]. In fact, that study suggested that peripheral homing and retention of CD8^+^ T cells in the respiratory tract is a mechanism to ensure an adequate number of memory T cells being available at the site of potential future respiratory tract infections.

Using in-vivo ^19^F-MRS we detected the donor cells in the area of the abdomen (corresponding mainly to the liver), the chest (corresponding mainly to the lungs and a portion of the liver) and the left flank (corresponding to the spleen and a portion of the liver) (Fig 8B). While MRS is most sensitive for ^19^F signal detection, it yields only limited spatial information. However, if sufficient ^19^F is brought into the target tissue, MRI is able to detect and image ^19^F-labeled SP and T_act_, as exemplified in Fig 9.

### SP and T cell behavior after IV injection

While ex-vivo high-resolution ^19^F-NMR was used to determine the true ^19^F atom content of the organs, flow cytometry was employed to determine the donor cell distribution in the animals. With this technique, the adoptively transferred cells were identified in the liver, lungs, and the spleen as with ^19^F-MRS, but flow cytometry also detected the donor cells in the tumor. As SP with a percentage of 0.06% ± 0.05% of donor cells (CD3^+^; CD8^+^; CD45.2^+^) measured by flow cytometry were detected in the liver by ^19^F-MRI, one would expect to also image T_act_ and T_OVA-act_ as they were observed in the organs at a considerably higher percentage than SP (i.e. 0.07% to 0.71%, for T_act_ 0.35% to 2.94% for T_OVA-act_ in the various organs; Fig 10A). However, ^19^F was detected by ^19^F-MRI in only one T_act_ treated mouse. This low rate of detection could be explained by the fact that following both antibody (against CD3 and CD28) and peptide stimulation, both T_act_ and T_OVA-act_ have the capacity to divide rapidly in-vivo. This proliferation of T cells in-vivo would then induce a subsequent dilution of the PFC content in the daughter cells. This dilution of ^19^F signal in the daughter cells can be verified in the present study by comparing the ex-vivo high resolution spectroscopy data to flow cytometry data.

The flow cytometry data also demonstrate that T_OVA-act_ have a better capacity than T_act_ and SP to infiltrate the different organs and the tumor. Both, T_act_ and T_OVA-act_ were produced from OT-1 mice and therefore, both T cells are expected to present predominantly the TCR that recognizes the OVA antigen. Nevertheless, flow cytometry in Fig 10A and 10B demonstrates a trend towards higher infiltration of tumors by T_OVA-act_. In line with the high presence of T_OVA-act_ in the liver, lungs, spleen, and tumor, they were detected by ^19^F-MRS but not at a ^19^F signal level that allowed for imaging. This is in contrast to SP, which are detectable by imaging even with their low presence in these organs. The SP represent a heterogeneous population. Besides T and B cells they contain dendritic cells and macrophages, both of which are phagocytic and can accumulate 10 to 1000 times more ^19^F compared to T cell populations [25]. Also, as these phagocyte populations are terminally differentiated cells, they do not divide, unlike activated T cells. ^19^F-MRI was performed 24h after adoptive cell transfer, A division cycle every 8 hours of the PCF-labeled activated T cells e.g. could already reduce the cellular ^19^F content by a factor of 8 at the time of imaging. Taken together, this could explain why despite the relatively high number of T_OVA-act_ and T_act_ detected by flow cytometry in the target organs, a diminished ^19^F loading was present in these organs, which limited their detection by ^19^F-MRS and ^19^F-MRI.

From these data, it might be speculated that non-dividing cells would be best for this type of tracking, such as SP or highly-differentiated T cells e.g. killer cells. Another alternative would be to inject a higher number of ^19^F-labeled cells to compensate for dilution according the cell division rate.

### Limitations of the study and strategies to improve the detection limit of ^19^F-labeled T cells in the tumor tissue

Possible ways to increase the sensitivity of the ^19^F-MRI method to detect PFC-labeled T cells in the tumor could be to: 1) increase the ^19^F-label content of the injected cells by further optimizing the in-vitro T cell PFC-labeling procedure, 2) enrich the proportion of ^19^F-labeled T cells in the injected cell populations (e.g. by sorting PFC-FITC labeled cells by flow cytometry), 3). use preferentially non-dividing T cells to minimize the label dilution effect caused by cell division (which was the most likely reason in our study preventing T cell detection in the tumors), 4) use modified PFCs with shorter T_1_ (e.g. by gadolinium-coupling), 5) exploit higher magnetic field strength, 6) develop high performance ^19^F coils, and 7) exploit emerging fast pulse sequences. For example, combining this ^19^F-MRI technique with compressed sensing could be advantageous with regard to shortening the acquisition time. Compressed sensing was already applied successfully for ^19^F-MRI by Zhong and co-workers, but this pulse sequence was not available on our 9.4T system [26].

A potential limitation of the study was the rather rigorous threshold of >200 SNR of the ^19^F-spectroscopic signal to proceed to ^19^F-MRI. As non-localized spectroscopy yields the entire signal of the volume within the coil, a spectroscopic signal below this threshold does not exclude the possibility for locally high ^19^F concentrations that would allow for ^19^F-MRI. This notion is supported by the liver ^19^F-images after PFC-labeled T_act_ injection, which demonstrate a non-uniform signal distribution in the liver (Fig 9B).

## Conclusions

Immune cells, including total SP and activated T cells can be successfully labeled in-vitro by ^19^F-PFC and the T cells maintain the capacity to detect and kill the tumor cells after ^19^F-labeling. They are detectable after IV administration by in-vivo coil-localized ^19^F-MRS in liver, lungs, and spleen. IV-injected SP can also be imaged by in-vivo ^19^F-MRI while this is more difficult for T cells. In particular, the proportion and/or ^19^F content of the injected ^19^F-labeled T cells was too low to allow for tumor imaging. Flow cytometry of liver, lungs, spleen, and tumor demonstrates a higher number of T_OVA-act_ and T_act_ than SP in these organs. The difficulty in reliably detecting ^19^F-labeled T_OVA-act_ and T_act_ in flow cytometry-positive organs by ^19^F-MRS could be explained by T_OVA-act_ and T_act_ proliferation, which would dilute the ^19^F signal in the daughter cells and thus, in the target organs. Non-dividing in-vitro ^19^F-labeled cell species appear most promising to be tracked by ^19^F-MRS and/or ^19^F-MRI.
